# Assessment of direct analysis in real time accurate mass spectrometry for the determination of triclosan in complex matrices

**DOI:** 10.1007/s00216-021-03591-2

**Published:** 2021-08-10

**Authors:** M. Cobo-Golpe, J. García-Martín, M. Ramil, R. Cela, I. Rodríguez

**Affiliations:** Department of Analytical Chemistry, Nutrition and Food Sciences, IAQBUS - Institute of Research on Chemical and Biological Analysis, Universidade de Santiago de Compostela, R/Constantino Candeira SN, 15782 Santiago de Compostela, Spain

**Keywords:** Ambient ionization, Triclosan, Direct analysis in real time (DART), Accurate mass spectrometry, Environmental samples, Matrix effects

## Abstract

In this work, the applicability of direct analysis in real time coupled to accurate mass spectrometry (DART-MS) to the quantitative determination of triclosan (TCS) in samples with increasing complexity, from personal care products to extracts from sewage, is investigated. In the first term, DART-MS spectra of TCS as free phenol and as derivatized species are characterized; thereafter, the effects of several instrumental variables in the detectability of TCS (i.e., temperature, solvent, and compound holder) are discussed. Under final selected conditions, TCS was determined from its [M-H]^−^ ions, without need of derivatization, attaining an instrumental limit of quantification of 5 ng mL^−1^, with a linear response range up to 1000 ng mL^−1^. Complex matrices, such as solid-phase extracts obtained from environmental water samples, moderately inhibited the ionization efficiency of TCS, with signal attenuation percentages in the range of 6 to 57%, depending on the sample type and on the concentration factor provided by the SPE procedure. The accuracy of results obtained by DART-MS was evaluated using liquid chromatography (LC) with MS detection; in both cases, a time-of-flight (TOF) MS instrument was employed for the selective determination of the [M−H]^−^ ions of TCS (*m*/*z* values 286.9439 and 288.9410) using a mass window of 20 ppm. DART-MS did not only provide enough sensitivity to detect the presence of TCS in environmental samples (raw and treated wastewater as well as freeze-dried sludge), but also measured concentrations matched those determined by LC-ESI-TOF-MS, with only slightly higher standard deviations. During analysis of personal care products, containing much higher concentrations of TCS in a less complex matrix, both techniques were equivalent in terms of accuracy and precision.

**Supplementary Information:**

The online version contains supplementary material available at 10.1007/s00216-021-03591-2.

**Figure:**
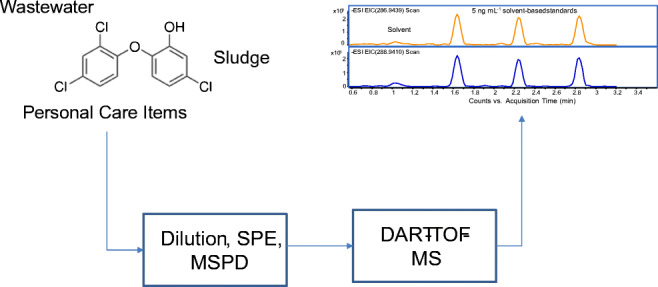
Graphical abstract

## Introduction

Ambient ionization techniques, such as direct analysis in real time (DART) [1, 2], have been introduced recently coupled to mass spectrometry for rapid analysis of different kinds of samples. These techniques provide quantitative and qualitative information of different sample components, avoiding the chromatographic separation step and, sometimes, also sample preparation. Despite these inherent benefits, in the case of DART, the ionization process is rather complex, involving several competitive routes, which depend on the properties of each compound (energy of ionization, functional groups, and thermal stability), the matrix components, and the ambient atmospheric molecules. What is generally accepted is that compounds are first desorbed from the sample and then ionized in the gas phase. In practice, the possibility to determine a certain compound in a complex chemical matrix, such as an environmental sample, depends on several parameters: the efficiency of the ionization source to generate ions from the neutral molecules, the matrix effects in the yield of the ionization process, and the selectivity (resolution) provided by the mass spectrometer in order to discriminate the signal of the analyte from those of other simultaneously generated ions during ionization [1, 2]. These characteristics make DART suitable for qualitative/quantitative analysis of bulk material components, usually containing a reduced number of different species in a simple matrix [3, 4]. However, the number of applications reporting the determination of trace compounds in complex matrices and/or in the concentrated extracts from environmental samples, i.e., wastewater, is still limited [5, 6].

Triclosan (TCS) is a synthetic antimicrobial agent which inhibits the activity of bacteria, viruses, and fungi [7]. It is widely used in various personal care products such as soaps, cosmetics, mouthwashes, and toothpaste [7, 8] and also in household items like toys, textiles, furniture, and kitchenware [9]. The above uses have led to the introduction of TCS in urban sewage and even in surface water due to its incomplete removal in wastewater treatment plants (STPs) [10–12]. The interaction of TCS with some water disinfectants has been correlated with the formation of halogenated disinfection by-products [13]. Moreover, its moderate lipophilic character, and chemical stability, have led to the distribution of this contaminant of concern in sewage sludge [14]. TCS has also been related to other environmental matrices, such as particulate matter or indoor dust [15]. Additionally to distribution studies, during the last years, the environmental risk assessment of triclosan has been widely investigated and harmful effects in aquatic plants [16] and fish [17] have been reported. More specifically, TCS has been found to be bioaccumulative in plants and earthworms [18–20], and correlated with the development of bacterial resistance [21, 22].

Thus, different analytical methodologies have been developed for the determination of triclosan in different products and environmental compartments. For aqueous samples, both solid-phase extraction [23, 24] and microextraction techniques [25–27] have been evaluated. Pressurized liquid extraction (PLE) [28] and matrix solid-phase dispersion (MSPD) [29] have been employed in the extraction of TCS from solid samples. In general, all these sample preparation techniques have been combined with an appropriate chromatographic technique, either gas chromatography or liquid chromatography, coupled to mass spectrometry. In some cases, the total protocol, sample preparation followed by chromatographic separation and detection, is rather time consuming. Furthermore, the use of a derivatization step in the case of GC-based techniques introduces an extra step in the analytical procedure.

To the best of our knowledge, to date, no thorough studies about triclosan determination by DART-MS have been carried out; therefore, the main objective of this work was to assess the possibilities of a DART source combined with a QTOF-MS instrument for the determination of this biocide in samples with different characteristics. These will include from personal care products, where the compound is added at concentrations of hundreds of milligrams per gram, to wastewater extracts and sewage sludge. In the case of environmental matrices, sample preparation protocols previously validated in combination with GC-MS/MS or LC-MS/MS have been used [30, 31]. Parameters affecting the detectability of TCS by DART-MS are optimized, and the accuracy of the results obtained during analysis of real samples is assessed using LC-ESI-QTOF-MS as reference technique.

## Material and methods

### Solvents, standards, and sorbents

Methanol (MeOH) (HPLC grade), acetonitrile, ethyl acetate, and formic acid were purchased from Merck (Darmstadt, Germany). Triclosan (TCS) and triclosan ^13^C_6_ (TCS-^13^C_6_) were provided by Toronto Research Chemicals Inc. (North York, ON, Canada). Acetic anhydride and the silylation agent N-methyl-N-(tert-butyldimethylsilyl)-trifluoroacetamide (MTBSTFA) were provided by Sigma-Aldrich (Milwaukee, WI, USA). Individual stock standards of TCS and TCS-^13^C_6_ were prepared in MeOH. Further dilutions were made in the same solvent, and stored at − 20 °C. Calibration standards for DART were prepared in ethyl acetate from methanol stocks.

OASIS HLB cartridges (60 mg) were obtained from Waters (Milford, MA, USA), C_18_ was provided by Agilent (Wilmington, DE, USA), diatomaceous earth was provided by VWR Chemicals (Leicestershire, England), and 10-mL polypropylene syringes were purchased from Becton Dickinson (Franklin Lakes, NJ, USA).

### Samples and sample preparation

Toothpaste and mouthwash samples were purchased in local supermarkets. Wastewater samples were collected in the effluent and influent of an urban sewage plant equipped with primary and secondary treatments. This plant receives the combined wastewater from a city of 125,000 inhabitants and also from a large hospital. Samples were passed through glass fiber filters and then stored at 4 °C until being analyzed. Sludge samples were collected from different STPs from Galicia (northwest of Spain) and freeze-dried after reception.

Mouthwash was simply diluted with methanol before analysis in order to bring TCS within the linear response range of DART-TOF-MS. Water samples were extracted by SPE using OASIS HLB cartridges (60 mg). Volumes of 200 and 400 mL were processed for influent and effluent samples, respectively. Samples were previously acidified at pH 3 and TCS-^13^C_6_ added before extraction. Elution of the cartridges was performed with 2 mL of ethyl acetate [30].

Sludge extraction was performed by MSPD. Briefly, approximately 500 mg of freeze-dried sludge, spiked with TCS-^13^C_6_, was dispersed over 2 g of C_18_ in a mortar and transferred to a polypropylene syringe containing 1 g of diatomaceous earth. The elution was carried out with 10 mL of ethyl acetate. This methodology was based on a generic MSPD extraction protocol developed in our group [31]. MSPD was also applied to the extraction of TCS from the toothpaste matrix. In this case, sample and dispersant (C18) masses were reduced to 0.2 and 1 g, respectively. The rest of the conditions were common to those employed for MSPD of freeze-dried sludge. Verification of the performance of both SPE and MSPD techniques rendered overall recoveries for wastewater and sludge higher than 88% (see Table S1).

### Equipment and determination conditions

#### DART-QTOF-MS

A DART-SVP ion source (IonSense Inc. Saugus, MA, USA, model SVPS-200) equipped with a linear rail was used in this research. This module was employed to hold either *Quick Strip* transmission sample cards, with a 12-position frame of stainless steel, or a *Dip it* accessory with 12 positions for glass capillaries, both allowing the analysis of liquid samples. The DART source was coupled to a QTOF-MS, Agilent 6520 model acquired from Agilent Technologies (Wilmington, DE, USA), through the commercial Vapur chamber, which reduces the entrance of helium or nitrogen in the high-vacuum region of the MS instrument. Helium was used as volatilization and ionization gas, and N_2_ was employed to refrigerate the source in the standby mode. In both cases, a flow rate of 2.5 mL min^−1^ was used. During method development, DART was operated in positive and negative ionization modes for native and derivatized TCS, applying a grid voltage of 350 V. The temperature of the source was set at 350 °C and the speed of the linear rail fixed at 0.3 mm s^−1^. Standards and sample extracts (from 1 to 4 μL) were deposited in the stainless-steel mesh of cards, or at the capillary tip, using a 10-μL syringe. In both cases, the solvent was allowed to evaporate for about 5 min, before attaching the holding material (steel mesh or glass capillaries) to the linear rail module.

The QTOF instrument operated in the extended dynamic range (2 GHz) mode with a resolution of 7800 (FWHM) measured for the ion at *m*/*z* 301.9981. Under final working conditions, TCS was quantified in negative ionization mode, as underivatized compound, applying a capillary voltage of 1000 V. The fragmentor voltage was set at 130 V. Accurate mass data were recorded in the range of *m*/*z* values from 70 to 1700, at a rate of 2 spectra s^−1^ (6767 scans are accumulated in each spectrum). Continuous recalibration of the *m*/*z* axis in the QTOF system was carried out with signals obtained for ions at *m*/*z* values of 89.0234 and 255.2324. These ions were generated from species (polyethylene glycol derivatives) existing in the atmosphere of the laboratory [32]. In the experiments performed operating the DART source in the positive mode, the recalibration ions were 135.1016 and 391.2843 corresponding to diethylene glycol monoethyl ether and bis (ethylhexyl) phthalate ionization, respectively. Both species are recognized as ubiquitous in indoor environments and provided signals with enough intensity for continuous re-calibration of the TOF-MS analyzer.

#### LC-QTOF-MS

In this case, a UPLC chromatograph (Agilent Infinity 1290) coupled to a second QTOF-MS system (Agilent 6550) equipped with an ESI ionization source was used. The system was operated in the 2 GHz mode, with a typical resolution (FWHM) of 19,500, at *m*/*z* 301.9981, almost double than that obtained with the other QTOF instrument combined with the DART source. The separation of TCS was performed in a C18 type column, acquired from Agilent (Zorbax Eclipse Plus C18, 50 mm × 2.1 mm, 1.8 μm), employing water (A) and acetonitrile (B) (both 0.1% formic acid) as mobile phases with a flow of 0.4 mL min^−1^. The gradient was as follows: 0–0.1 min (90% A), 6–7 min (0% A), and 7.1–10 min (90% A). The column temperature was fixed at 40 °C. The quantification ions were 286.9439 and 288.9410 for TCS and 292.9618 for TCS-^13^C_6_. The isolation window was 20 ppm and the injection volume, 2 μL.

### Recovery assessment and sample quantification

The calibration range employed in the DART-QTOF-MS and UPLC-ESI-QTOF-MS systems was from 10 to 1000 ng mL^−1^. The internal standard (IS, TCS-^13^C_6_) was kept at 100 ng mL^−1^. Peak areas for the quantification ions were divided by the signal for the IS and plotted versus the concentration. The levels of TCS in the processed samples were calculated considering the concentrations measured in the calibration plots obtained for solvent-based standards, the mass (volume) of sample employed in the corresponding sample preparation approach, and the volume of extracts (2 and 10 mL in the case of SPE and MSPD extracts, respectively). TCS-^13^C_6_ was added to samples at the equivalent level to that used in calibration standards: 100 ng mL^−1^ referred to the final extract.

Matrix effects were evaluated by preparing standards in ethyl acetate and sample extracts (wastewater or sludge) in the same solvent. No IS correction was carried out. The slopes of the calibration curves for matrix-matched standards were normalized (divided) with those corresponding to solvent-based standards. Values lower than 100% indicate signal suppression and values higher than 100% correspond to an increase of the DART ionization efficiency for sample extracts versus solvent-based standards.

The accuracy of the results obtained by DART-QTOF-MS was evaluated using UPLC-ESI-QTOF-MS as reference technique, applied to the determination of the TCS concentration in the extracts obtained for non-spiked fractions of different samples. In the case of SPE and MSPD sample preparation, extracts were exchanged to MeOH before analysis. The aim of such comparison was to assess whether the measurements at the DART source are affected by isobaric interferences (ions with similar *m*/*z* ratios, not separated by the TOF mass analyzer) proceeding from the ionization of other analytes present in the sample.

## Results and discussion

### DART-MS spectra of TCS

Previous studies [33] have demonstrated that certain phenols are fragmented in the DART source, likely during desorption, rendering ions with lower *m*/*z* ratios than the parent compound, leading to high limits of detection. In the case of bisphenols, this drawback was overcome considering compounds acetylation. Thus, the efficiency of TCS ionization was investigated as free compound and after acetylation and silylation (as dimethyl, tert-butyl silyl derivative), operating the TOF-MS instrument in positive and negative acquisition modes. TCS derivatives were prepared as described elsewhere [15, 34].

In Table 1, the identities and the *m*/*z* ratios of the main ions observed in the spectra of TCS without a derivatization step, or after derivatization, are shown. It is worth noticing that the completeness of derivatization reactions was assessed with the EI-MS spectra of TCS as silylated (dimethyl, tert-butyl silyl derivative) or acetylated species; see Supplementary information, Fig. S1 and S2. As regards the underivatized compound, no signal was noticed when operating the MS system in the positive mode, while the typical isotopic pattern of a trichlorinated phenolate [M-H]^−^ was noticed in the negative mode. The silylated derivative was found to be unstable during the volatilization-ionization process; thus, the only cluster of ions observed was those associated to the deprotonated compound in the negative acquisition mode. Finally, the acetylated form led to an adduct with ammonium, observed when operating the MS spectrometer in the positive mode. Thus, conversely to the silylated forms, acetyl derivatives remain stable in the DART source leading to adducts with NH_4_^+^ ions, as it has been previously reported for bisphenol species [33].

**Table 1:** Identities of base ions in the DART spectra of TCS as a function of derivatization technique and polarity

Derivatization	Ionization	Ion	*m*/*z*
None	−	[M − H]^−^	286.9439
Acetylation	+	[M + C_2_OH_2_ + NH_4_]^+^	347.9956
Silylation	−	[M − H]^−^	286.9439

The comparison of responses obtained for [M − H]^−^ ions, observed for free TCS, and those for the adduct of the acetylated derivative with ammonium [M + C_2_OH_2_ + NH_4_]^+^, reflected a significantly higher response for free TCS. So, derivatization was no longer considered.

### Instrumental parameters of the DART source

One of the variables affecting the efficiency of the volatilization and ionization process at DART is the temperature of the excited helium atom current that incises over the sample holder. At the same time, the holder (Quick Strip or Dip It, Figs. S3–S6), where the species is deposited, might also affect the signal intensity. To assess the effect of the latter parameter, responses obtained for the [M − H]^−^ ion of solvent-based standards with concentrations comprised between 50 and 400 ng mL^−1^ were evaluated. In both groups of experiments, the volume of standard deposited in the metal grid (Quick Strip Cards, Figs. S3–S4) or on the glass capillaries tip (Dip-It system, Figs. S5–S6) was 2 μL. Once the solvent was completely evaporated, the modules were incorporated to the mobile rail.

**Fig. 1:**
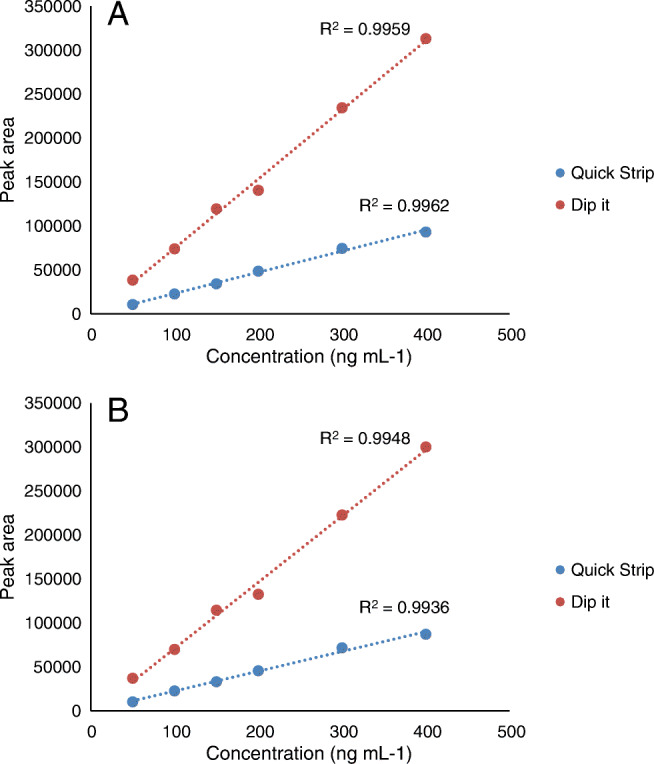
Comparison of slopes obtained using different holders for standards (*n* = 2 replicates per calibration level) of TCS in ethyl acetate. Quantification ions *m*/*z* 286.9439 (**A**) and *m*/*z* 288.9410 (**B**). Mass extraction window 20 ppm

Figure 1 shows the obtained results (peak areas) for solvent-based standards with different concentrations from 50 to 400 ng mL^−1^. The highest slopes were obtained with the Dip-It system. Likely, the metal mesh used in the transmission grids reduces the effective energy over the sample. For a desorption temperature of 350 °C, the obtained responses with the glass capillaries were three times higher than those obtained with the metal grid holder. Another drawback observed with the metal grid is that the holder interrupts the helium atom flow that reaches the inlet orifice of the vapor chamber between sample spots; thus, there is not a continuous flow of background ions (some of them used to guarantee the stability of mass calibration) entering the MS spectrometer. Thus, the Dip-It system was selected for the study.

**Fig. 2:**
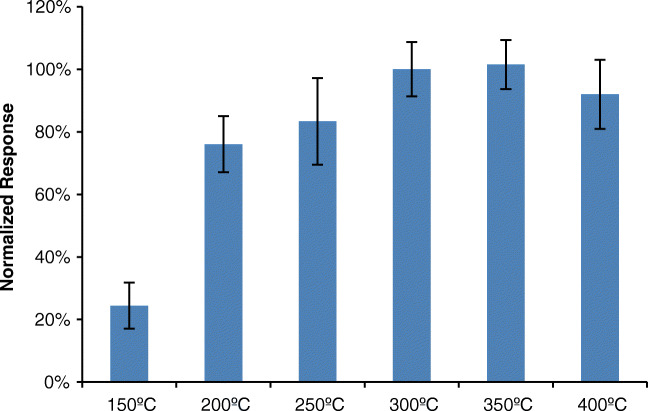
Variations in the response of TCS as a function of DART temperature, *n* = 3 replicates

In order to choose the optimum working temperature, replicated samples were measured at different temperatures. In this case, a standard of 1 μg mL^−1^ of TCS was used and replicate determinations (*n* = 3), at temperatures between 150 and 400 °C, were performed. As it can be observed in Fig. 2, the response increases with the temperature till reaching a plateau at 300–350 °C. Thus, it was decided to operate at 350 °C for the rest of the study. Higher temperatures could lead to the thermal degradation of the compound.

### Performance of DART-TOF-MS detection for TCS

**Fig. 3:**
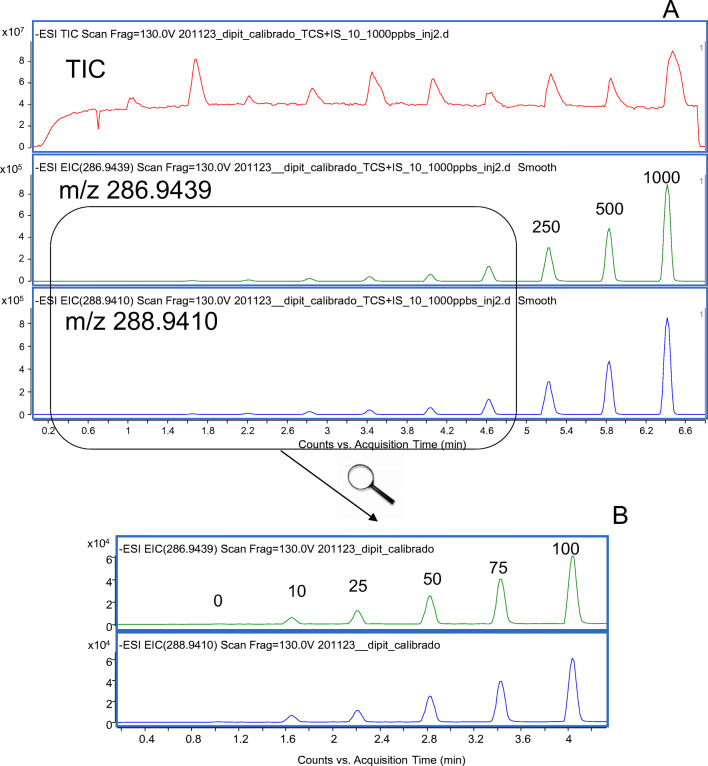
**A** Plots of DART-QTOF-MS responses (negative mode) vs time obtained for a series of standards in ethyl acetate in the range of concentrations from 10 to 1000 ng mL^−1^. Extraction ions, 286.9439 and 288.9410; mass window, 20 ppm. **B** Enlargement of the region 0.3–4.2 min

The linearity of the system was investigated in the range of concentrations from 10 to 1000 ng mL^−1^. Calibration curves were constructed for the ions with *m*/*z* 286.9439 and 288.9410, employing a mass extraction window of 20 ppm. As TCS is a trichlorinated species, the response ratio between both ions was close to the unit (experimental ratios were between 1.05 and 1.07, depending on the employed holder). This relationship must be constant during the real sample analysis. In both cases, the determination coefficients (*R*^2^) were above 0.998 in the range of concentrations of two orders of magnitude. Figure 3 shows the plots of the DART-MS response vs time obtained for the quantification ions of TCS. The first observed signal (c.a. 1 min in the plot) corresponds to a calibration blank containing just the IS. The whole time required to obtain a calibration curve (9 calibration levels and 1 blank) was 6.6 min.

In Table 2, a comparison of slopes and *R*^2^ values for calibrations in the same concentration range, and varying the solvent employed for the preparation of standards, is shown. As it can be noticed, the type of solvent affected the calibration curve slopes, ethyl acetate being the solvent which showed the highest sensitivity. Probably, changes in the obtained responses as a function of the solvent are related with TCS distribution on the surface of glass tips of the Dip-It module after solvent evaporation. Whatever the exact reason, ethyl acetate was maintained as calibration solvent, unless otherwise stated.

**Table 2:** Normalized slopes and determination coefficients (*R*^2^) obtained with a calibration of TCS without internal standard and different solvents; quantification ion, 286.9439. (Range 10–1000 μg L^−1^, 9 calibration levels)

Solvent	Normalized slopes	*R*^2^
Ethyl acetate	1.000 ± 0.016	0.9983
MeOH	0.687 ± 0.015	0.9965
Chloroform	0.750 ± 0.027	0.9913
Ultrapure water	0.452 ± 0.010	0.9981

The instrumental limit of quantification (LOQ) of the DART-QTOF-MS was estimated from the signal to noise ratio obtained for the standard with the lowest concentration in the calibration. Thus, the minimum level of concentration that yields peaks with a signal to noise ratio of 10 was evaluated. The relationship between both quantification ions of TCS was kept at 1.0 ± 15%. In general, an instrumental LOQ of 5 ng mL^−1^ for a volume of 2 μL (Fig. S7) has been established.

The presence of organic and inorganic compounds in real samples (or in their extracts) can give rise to competence processes during TCS ionization. These processes could cause a significant attenuation of the TCS ionization in the DART source. Changes in the efficiency of TCS ionization could be expected to be more relevant than in LC-ESI-MS, since, in the absence of a chromatography separation step, all species present in the sample (in the case of environmental samples in the corresponding extract) are ionized simultaneously. This will produce a significant increase of the procedural LOQ, reducing the applicability of the methodology to real-sample analysis.

**Fig. 4:**
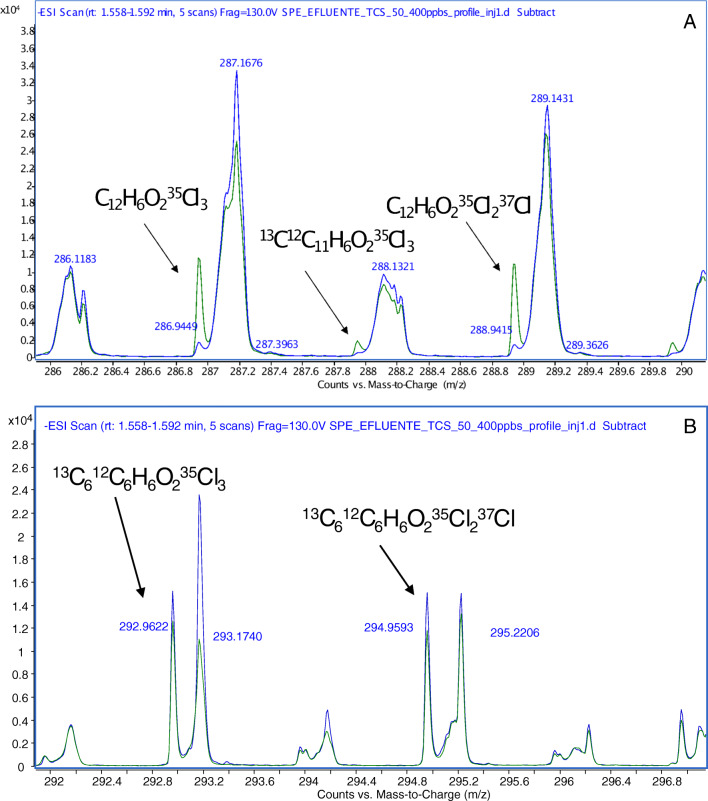
Details of the mass spectra for TCS (**A**) and TCS-^13^C_6_ (**B**) in a spiked treated wastewater extract (50 ng mL^−1^, green plot) versus a non-spiked extract from the same matrix (blue plot)

In this study, the comparison of slopes for SPE extracts of ultrapure water, river water, and treated and raw wastewater, after a 200-fold concentration factor by SPE (100-fold for raw wastewater), was carried out. Table 3 compiles the data corresponding to curve slopes, their determination coefficients (*R*^2^), and the attenuation percentage in the signal for the real samples. The highest attenuation (around 56%) was observed for the influent sample. The plots of the DART-MS response vs time obtained for a spiked treated wastewater sample (50–400 ng mL^−1^ addition levels) are compiled in Fig. S8. Figure 4 shows a region of the spectra observed for the non-spiked and the spiked extracts (addition level 50 ng mL^−1^) of a treated wastewater after 200-fold concentration by SPE. As observed, both spectra showed saturated bands with a baseline width of 0.2 Da at nominal *m*/*z* values of 287, 288, and 289 Da. Consequently, it becomes impossible to quantify any species rendering ions within those bands. The successful selective determination of TCS, and the isotopically labeled analogue TCS-^13^C_6_, is associated to the negative mass defect of this compound (ca. 56 and 59 mDa for C_12_H_6_O_2_^35^Cl_3_ and C_12_H_6_O_2_^35^Cl_2_^37^Cl ions, respectively); thus, the deprotonated molecular ions of this species show *m*/*z* ratios in a relatively clean spectral region. Thus, even the relatively poor resolution of masses provided by the first-generation TOF instrument combined with the DART source in this study permitted to discriminate TCS ions from co-extracted wastewater compounds.

**Table 3:** Slopes and determination coefficients (*R*^2^) for matrix-matched calibration solutions prepared with SPE extracts from different water samples

Sample	Quantification ion	Slope	Intercept	*R*^2^ (10–400 ng mL^−1^, *n* = 6 levels)	% Signal attenuation
Ultrapure water	286.9439	800.5	200	0.9869	–
	288.9399	761.9	190	0.9874	–
River water	286.9439	751.4	412	0.9974	6.1 ± 0.2%
	288.9399	712.9	376	0.9927	6.4 ± 0.3%
Raw wastewater	286.9439	354.4	2610	0.9789	56 ± 3%
	288.9399	326.3	2273	0.9743	57 ± 3%
Treated wastewater	286.9439	558.8	3110	0.9951	30 ± 1%
	288.9399	533.4	2515	0.9961	30 ± 1%

In summary, considering the instrumental LOQs of DART-MS, the concentration factor provided by SPE (100- and 200-fold for raw and treated wastewater), and moderate attenuation in the efficiency of TCS ionization in the case of wastewater extracts, the estimated LOQs of the overall procedure are in the range of 100 to 35 ng L^−1^ for raw and treated wastewater, respectively. In the case of sludge, without considering signal attenuation effects, LOQ is estimated in the range of 100 ng g^−1^. Such values might be low enough to permit the quantification of TCS in these environmental matrices.

### Analysis of real samples

The accuracy of the DART-QTOF-MS system was investigated through the analysis of non-spiked samples, considering matrices with different complexity, and using UPLC-QTOF-MS as reference technique. In both cases, TCS-^13^C_6_ was used as surrogate standard added to samples before extraction (dilution in the case of mouthwashing formulations), and the concentration of TCS was estimated against solvent-based standards. In DART-MS, the only employed identification parameter was the ratio between responses for ions at *m*/*z* 286.9439 and 288.9410. When using UPLC-ESI-TOF-MS, retention time (window 0.1 min) was also considered for a positive identification. Pairs of wastewater samples (grab sampling) were obtained from the same STP at the end of 2020, while freeze-dried sludge samples correspond to four different STPs. Table 4 compiles the TCS concentration values and the standard deviation for the personal care product samples (mouthwash and toothpaste) and the environmental processed samples (raw and treated wastewater and sludge). In all the cases, data correspond to triplicate analysis of same extracts (samples). When required (personal care products) samples, or sample extracts, were diluted to accommodate the levels of TCS between 10 and 1000 ng mL^−1^. In the case of personal care product samples, there is a good coincidence between the values (average concentrations with their standard deviations) obtained with both techniques. It is obvious that the complexity of DART-MS spectra for this kind of samples is significantly lower than that shown in Fig. 4 for SPE extracts of wastewater, which turns in a better precision (Fig. S9). On the other hand, for the wastewater and sludge samples, the concordance between average values is also acceptable. Regarding repeatability, DART-TOF-MS renders worse results (higher dispersion). However, a suitable estimation of the TCS levels in those samples has been provided.

**Table 4:** Concentration of TCS in personal care products and environmental samples obtained through analysis of obtained extracts by DART-TOF-MS and UPLC-ESI-TOF-MS, *n* = 3 replicates

Sample	TCS concentration (%, ng L^−1^ or ng g^−1^)	
	DART-QTOF-MS	UPLC-QTOF-MS
Mouthwash	0.20 ± 0.02%	0.21 ± 0.01%
Toothpaste	0.28 ± 0.07%	0.28 ± 0.01%
Effluent 1(26/11/20)	67 ± 13 ng/L	67 ± 3 ng/L
Effluent 2 (04/12/20)	75 ± 10 ng/L	66 ± 2 ng/L
Effluent 3 (09/12/20)	47 ± 10 ng/L	57 ± 1 ng/L
Influent 1 (26/11/20)	237 ± 20 ng/L	196 ± 19 ng/L
Influent 2 (04/12/20)	115 ± 10 ng/L	94 ± 2 ng/L
Influent 3 (09/12/20)	120 ± 10 ng/L	103 ± 5 ng/L
Sludge 1	136 ± 5 ng/g	162 ± 4 ng/g
Sludge 2	1004 ± 50 ng/g	981 ± 49 ng/g
Sludge 3	753 ± 98 ng/g	733 ± 44 ng/g
Sludge 4	324 ± 52 ng/g	426 ± 4 ng/g

Data provided in Table 4 confirm that TCS is still present in the urban STPs despite the imposed restrictions to its use during the last years. On the other hand, the possibility of using a DART-QTOF-MS system to measure TCS at the concentration level existing in real samples without the spectral signals for TCS being affected is highlighted.

## Conclusions

DART-MS allows the sensitive determination of TCS from its deprotonated ions, without the need of any derivatization reaction. The achieved instrumental LOQs are mainly affected by the temperature of the DART source and by the device employed to hold TCS solutions between the tip of the DART and the entrance of the Vapur interface connected to the MS spectrometer. For the particular application considered in the current research, the use of glass capillaries was advantageous to metallic frames. The level of spectral interferences observed when analyzing complex extracts from environmental samples was relatively low. The typical mass defect for chlorine isotopes is responsible for the selective determination of TCS. The signal suppression for complex environmental extracts was in the range of that obtained frequently by LC-ESI-MS. Therefore, in combination with the use of isotopically labeled analogue, DART-TOF-MS provides acceptable results in terms of precision and accuracy to determine TCS levels not only in personal care products but also in environmental samples.

## Supplementary information

ESM 1 (DOCX 1595 kb)
