# Supplementary material for: Assessment of direct analysis in real time accurate mass spectrometry for the determination of triclosan in complex matrices
Source: Anal Bioanal Chem. 2021 Aug 10;413(25):6355–64. doi: 10.1007/s00216-021-03591-2 (PMC8487875; doi:10.1007/s00216-021-03591-2)
Supplement: Supplementary file 1 — (DOCX 1595 kb) [file 216_2021_3591_MOESM1_ESM.docx]

Supplementary information to manuscript:

**Assessment of Direct Analysis in Real Time accurate Mass Spectrometry for the Determination of Triclosan in Complex Matrices**

Cobo Golpe, M.; García-Martín, J.; Ramil, M.*; Cela, R.; Rodríguez, I.

*Department of Analytical Chemistry, Nutrition and Food Sciences, IAQBUS - Institute of Research on Chemical and Biological Analysis, Universidade de Santiago de Compostela, R/Constantino Candeira SN, 15782 Santiago de Compostela, Spain*

e-mail:maria.ramil@usc.es

**TABLES:**

Table S1. Recoveries of SPE and MSPD for raw wastewater (addition level 500 ng/L) and freeze-dried sludge (addition level 500 ng/g), n=3 replicates.

| Sample | Mean recovery (%) | SD |
| --- | --- | --- |
| Raw wastewater | 98 | 2 |
| Freeze-dried sludge | 88 | 13 |

**FIGURES:**

Figure S1. EI-MS spectrum of acetylated TCS, *m/z* range 40-380.

Figure S2. EI-MS spectrum of TCS as dimethyl tert.-butyl silyl derivative, *m/z* range 40- 440.

Figure S3. Metal grid (Quick Strip Card) picture


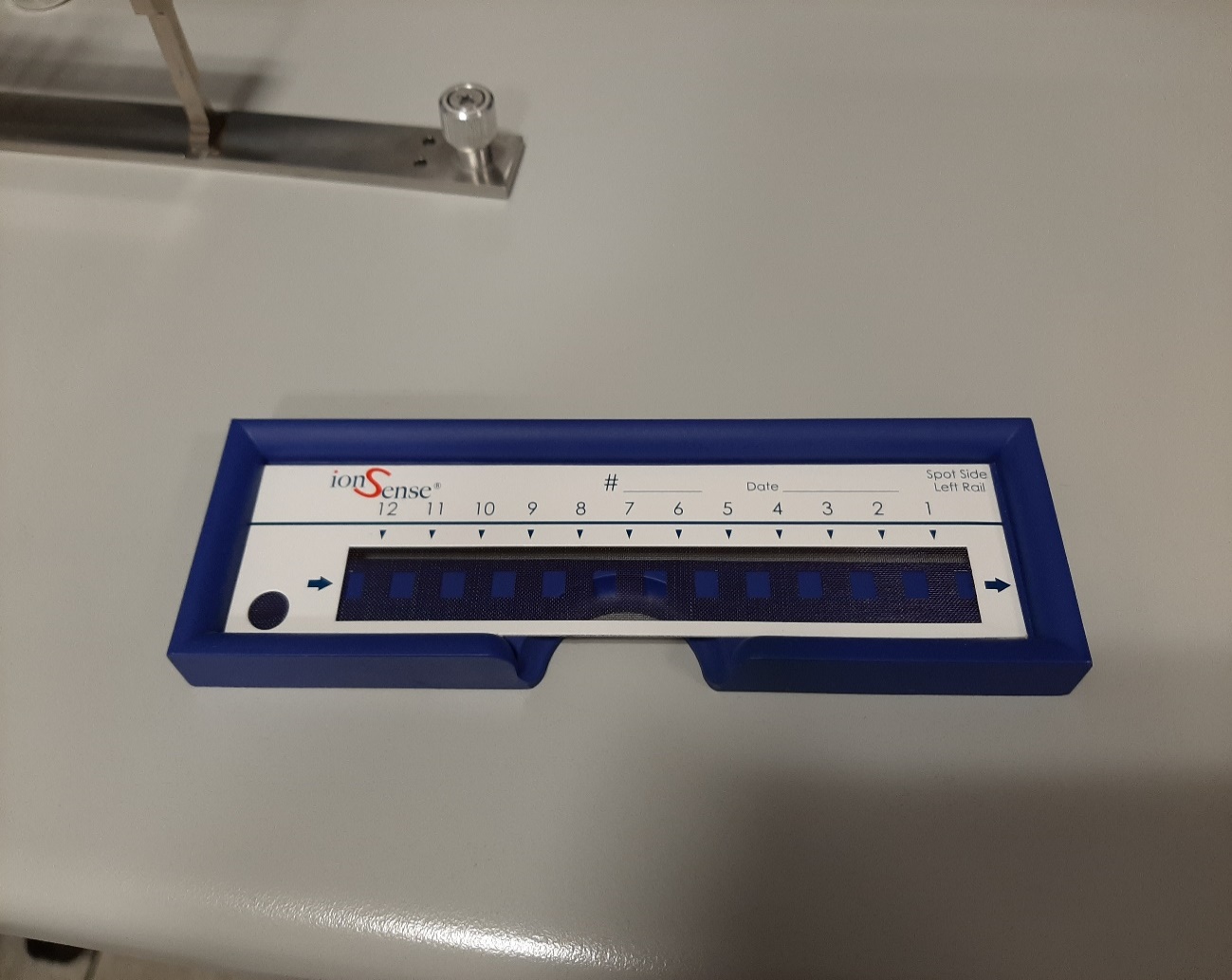


Figure S4.Quick Strip mobile rail and holder.

*
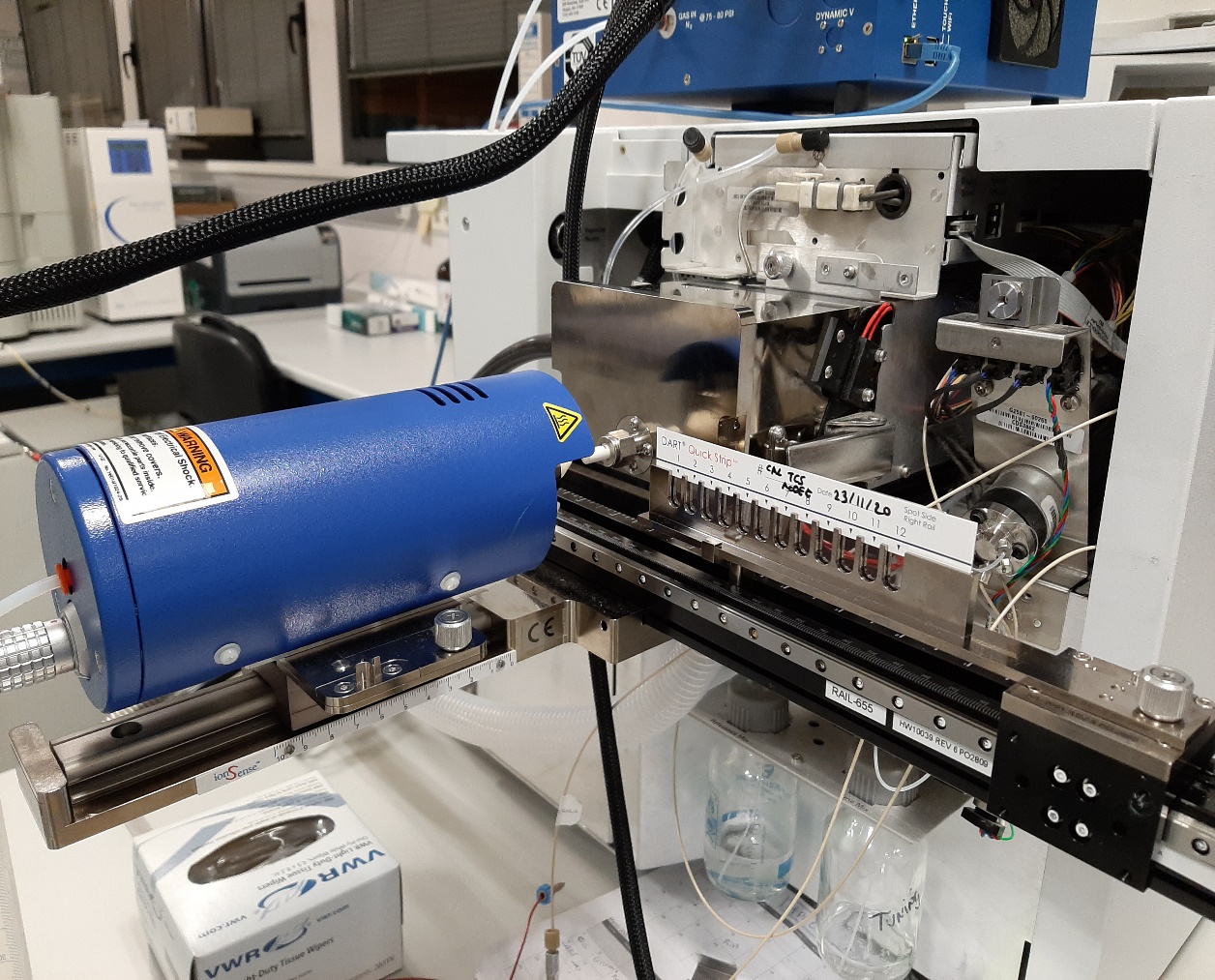
*

*
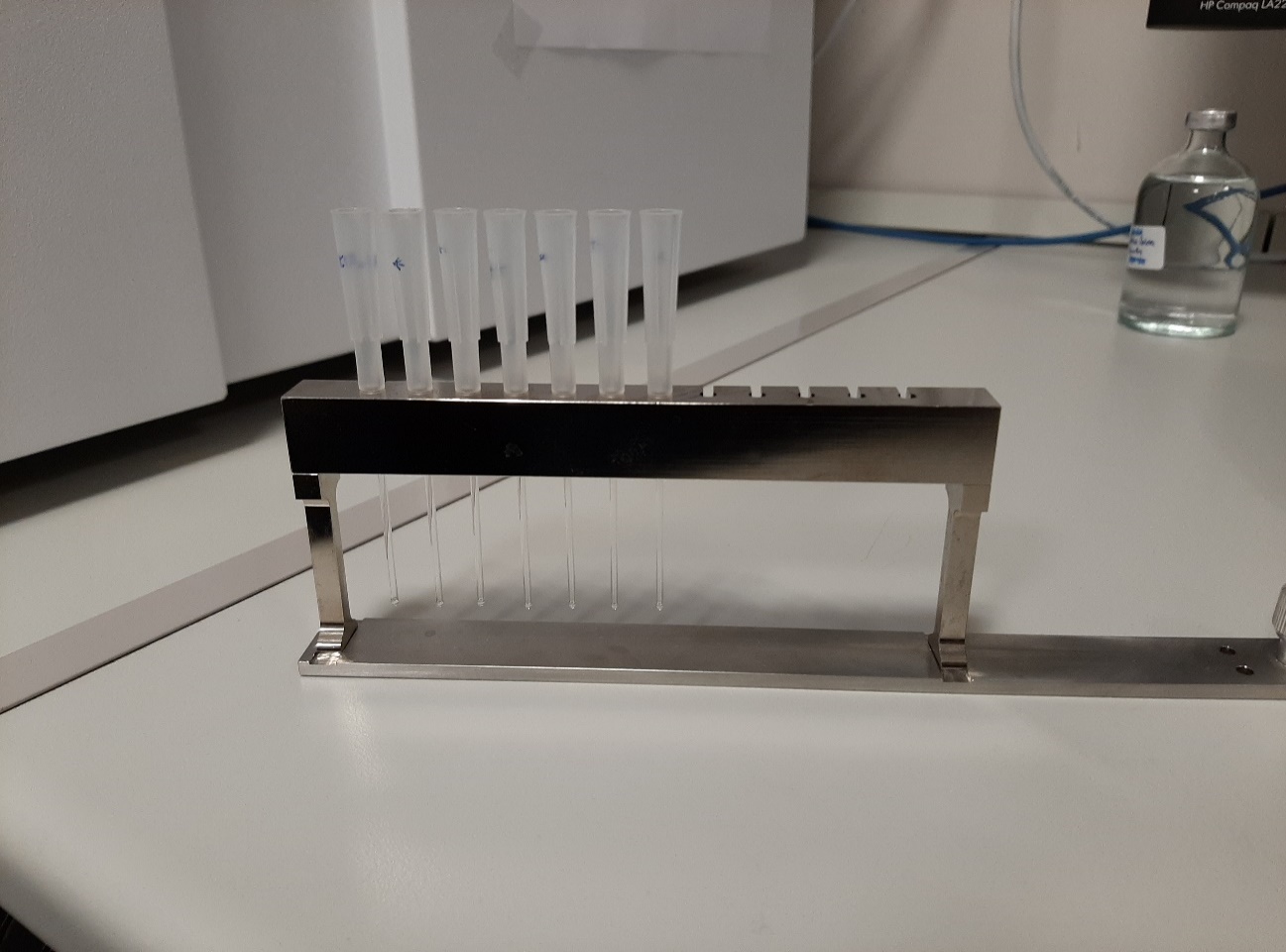
*Figure S5: Glass capillaries configuration (Dip-It)

Figure S6: Dip-It mobile rail and holder.

*
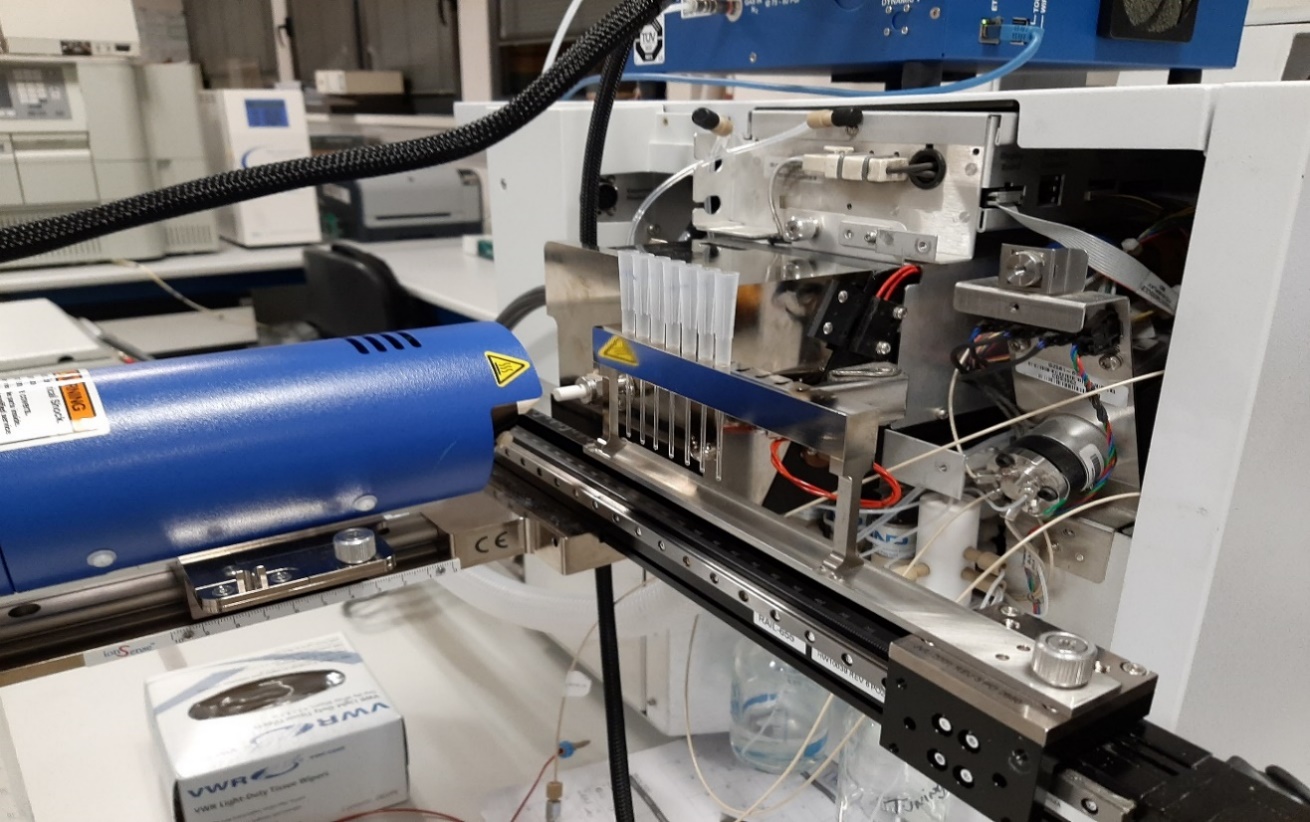
*

Figure S7: Plot of DAR-TOF-MS responses versus time for TCS standards in ethyl acetate. Blank and 3 replicates of a 5 ng mL^-1^ standard. Quantification ions: m/z 286.9439 (A) and 288.9410 (B).

A

B

Figure S8: TIC and EIC (286.9439 and 288.9410) responses versus acquisition time forTCS in spiked extracts from treated wastewater (50-400 ng mL^-1^ )

*m/z* 286.9439

*m/z* 288.9410

0

50

100

75

200

300

400

Fig. S9. A, profile spectrum obtained for a personal care sample (diluted mouth washing solution) by DART-TOF-MS. B, detail of the region corresponding to the [M-H]^-^ ions of TCS.
